# Supplementary material for: HIVprotI: an integrated web based platform for prediction and design of HIV proteins inhibitors
Source: J Cheminform. 2018 Mar 9;10:12. doi: 10.1186/s13321-018-0266-y (PMC5845081; doi:10.1186/s13321-018-0266-y)
Supplement: Supplementary file 2 — Additional file 2. Source code of HIVProtI web server. [file 13321_2018_266_MOESM2_ESM.zip › HIVprotI_Source-code/hivprotI/weka-3-6-6/doc/weka/classifiers/meta/AttributeSelectedClassifier.html]

AttributeSelectedClassifier


---


|  |  |  |
| --- | --- | --- |
| **Overview** \| **Package** \| **Class** \| **Tree** \| **Deprecated** \| **Index** \| **Help** \| **Weka's home** | |  |
| **PREV CLASS**   **NEXT CLASS** | **FRAMES**    **NO FRAMES**     **All Classes** |  |
| SUMMARY: NESTED \| FIELD \| CONSTR \| METHOD | DETAIL: FIELD \| CONSTR \| METHOD |  |


---


## weka.classifiers.meta Class AttributeSelectedClassifier

```
java.lang.Object
  weka.classifiers.Classifier
      weka.classifiers.SingleClassifierEnhancer
          weka.classifiers.meta.AttributeSelectedClassifier
```

**All Implemented Interfaces:**: java.io.Serializable, java.lang.Cloneable, AdditionalMeasureProducer, CapabilitiesHandler, Drawable, OptionHandler, RevisionHandler, WeightedInstancesHandler

---

``` public class AttributeSelectedClassifier extends SingleClassifierEnhancer implements OptionHandler, Drawable, AdditionalMeasureProducer, WeightedInstancesHandler ```

Dimensionality of training and test data is reduced by attribute selection before being passed on to a classifier.


Valid options are:

```
 -E <attribute evaluator specification>
  Full class name of attribute evaluator, followed
  by its options.
  eg: "weka.attributeSelection.CfsSubsetEval -L"
  (default weka.attributeSelection.CfsSubsetEval)
```

```
 -S <search method specification>
  Full class name of search method, followed
  by its options.
  eg: "weka.attributeSelection.BestFirst -D 1"
  (default weka.attributeSelection.BestFirst)
```

```
 -D
  If set, classifier is run in debug mode and
  may output additional info to the console
```

```
 -W
  Full name of base classifier.
  (default: weka.classifiers.trees.J48)
```

```
 Options specific to classifier weka.classifiers.trees.J48:
```

```
 -U
  Use unpruned tree.
```

```
 -C <pruning confidence>
  Set confidence threshold for pruning.
  (default 0.25)
```

```
 -M <minimum number of instances>
  Set minimum number of instances per leaf.
  (default 2)
```

```
 -R
  Use reduced error pruning.
```

```
 -N <number of folds>
  Set number of folds for reduced error
  pruning. One fold is used as pruning set.
  (default 3)
```

```
 -B
  Use binary splits only.
```

```
 -S
  Don't perform subtree raising.
```

```
 -L
  Do not clean up after the tree has been built.
```

```
 -A
  Laplace smoothing for predicted probabilities.
```

```
 -Q <seed>
  Seed for random data shuffling (default 1).
```

**Version:**
:   $Revision: 1.26 $

**Author:**
:   Mark Hall (mhall@cs.waikato.ac.nz)

**See Also:**: Serialized Form

---

**Field Summary**

**Fields inherited from interface weka.core.Drawable**

`BayesNet, Newick, NOT_DRAWABLE, TREE`


**Constructor Summary**

`AttributeSelectedClassifier()`
  
          Default constructor.


| **Method Summary** | |
| --- | --- |
| `void` | `buildClassifier(Instances data)`             Build the classifier on the dimensionally reduced data. |
| `double[]` | `distributionForInstance(Instance instance)`             Classifies a given instance after attribute selection |
| `java.util.Enumeration` | `enumerateMeasures()`             Returns an enumeration of the additional measure names |
| `java.lang.String` | `evaluatorTipText()`             Returns the tip text for this property |
| `Capabilities` | `getCapabilities()`             Returns default capabilities of the classifier. |
| `ASEvaluation` | `getEvaluator()`             Gets the attribute evaluator used |
| `double` | `getMeasure(java.lang.String additionalMeasureName)`             Returns the value of the named measure |
| `java.lang.String[]` | `getOptions()`             Gets the current settings of the Classifier. |
| `java.lang.String` | `getRevision()`             Returns the revision string. |
| `ASSearch` | `getSearch()`             Gets the search method used |
| `java.lang.String` | `globalInfo()`             Returns a string describing this search method |
| `java.lang.String` | `graph()`             Returns graph describing the classifier (if possible). |
| `int` | `graphType()`             Returns the type of graph this classifier represents. |
| `java.util.Enumeration` | `listOptions()`             Returns an enumeration describing the available options. |
| `static void` | `main(java.lang.String[] argv)`             Main method for testing this class. |
| `double` | `measureNumAttributesSelected()`             Additional measure --- number of attributes selected |
| `double` | `measureSelectionTime()`             Additional measure --- time taken (milliseconds) to select the attributes |
| `double` | `measureTime()`             Additional measure --- time taken (milliseconds) to select attributes and build the classifier |
| `java.lang.String` | `searchTipText()`             Returns the tip text for this property |
| `void` | `setEvaluator(ASEvaluation evaluator)`             Sets the attribute evaluator |
| `void` | `setOptions(java.lang.String[] options)`             Parses a given list of options. |
| `void` | `setSearch(ASSearch search)`             Sets the search method |
| `java.lang.String` | `toString()`             Output a representation of this classifier |

**Methods inherited from class weka.classifiers.SingleClassifierEnhancer**

`classifierTipText, getClassifier, setClassifier`

**Methods inherited from class weka.classifiers.Classifier**

`classifyInstance, debugTipText, forName, getDebug, makeCopies, makeCopy, setDebug`

**Methods inherited from class java.lang.Object**

`equals, getClass, hashCode, notify, notifyAll, wait, wait, wait`

**Constructor Detail**

### AttributeSelectedClassifier

```
public AttributeSelectedClassifier()
```

:   Default constructor.


**Method Detail**

### globalInfo

```
public java.lang.String globalInfo()
```

:   Returns a string describing this search method

    :   **Returns:**: a description of the search method suitable for displaying in the explorer/experimenter gui

---


### listOptions

```
public java.util.Enumeration listOptions()
```

:   Returns an enumeration describing the available options.

    :   **Specified by:**: `listOptions` in interface `OptionHandler` **Overrides:**: `listOptions` in class `SingleClassifierEnhancer`
    :   **Returns:**: an enumeration of all the available options.

---


### setOptions

```
public void setOptions(java.lang.String[] options)
                throws java.lang.Exception
```

:   Parses a given list of options.


    Valid options are:

    ```
     -E <attribute evaluator specification>
      Full class name of attribute evaluator, followed
      by its options.
      eg: "weka.attributeSelection.CfsSubsetEval -L"
      (default weka.attributeSelection.CfsSubsetEval)
    ```

    ```
     -S <search method specification>
      Full class name of search method, followed
      by its options.
      eg: "weka.attributeSelection.BestFirst -D 1"
      (default weka.attributeSelection.BestFirst)
    ```

    ```
     -D
      If set, classifier is run in debug mode and
      may output additional info to the console
    ```

    ```
     -W
      Full name of base classifier.
      (default: weka.classifiers.trees.J48)
    ```

    ```
     Options specific to classifier weka.classifiers.trees.J48:
    ```

    ```
     -U
      Use unpruned tree.
    ```

    ```
     -C <pruning confidence>
      Set confidence threshold for pruning.
      (default 0.25)
    ```

    ```
     -M <minimum number of instances>
      Set minimum number of instances per leaf.
      (default 2)
    ```

    ```
     -R
      Use reduced error pruning.
    ```

    ```
     -N <number of folds>
      Set number of folds for reduced error
      pruning. One fold is used as pruning set.
      (default 3)
    ```

    ```
     -B
      Use binary splits only.
    ```

    ```
     -S
      Don't perform subtree raising.
    ```

    ```
     -L
      Do not clean up after the tree has been built.
    ```

    ```
     -A
      Laplace smoothing for predicted probabilities.
    ```

    ```
     -Q <seed>
      Seed for random data shuffling (default 1).
    ```

    :   **Specified by:**: `setOptions` in interface `OptionHandler` **Overrides:**: `setOptions` in class `SingleClassifierEnhancer`
    :   **Parameters:**: `options` - the list of options as an array of strings **Throws:**: `java.lang.Exception` - if an option is not supported

---


### getOptions

```
public java.lang.String[] getOptions()
```

:   Gets the current settings of the Classifier.

    :   **Specified by:**: `getOptions` in interface `OptionHandler` **Overrides:**: `getOptions` in class `SingleClassifierEnhancer`
    :   **Returns:**: an array of strings suitable for passing to setOptions

---


### evaluatorTipText

```
public java.lang.String evaluatorTipText()
```

:   Returns the tip text for this property

    :   **Returns:**: tip text for this property suitable for displaying in the explorer/experimenter gui

---


### setEvaluator

```
public void setEvaluator(ASEvaluation evaluator)
```

:   Sets the attribute evaluator

    :   **Parameters:**: `evaluator` - the evaluator with all options set.

---


### getEvaluator

```
public ASEvaluation getEvaluator()
```

:   Gets the attribute evaluator used

    :   **Returns:**: the attribute evaluator

---


### searchTipText

```
public java.lang.String searchTipText()
```

:   Returns the tip text for this property

    :   **Returns:**: tip text for this property suitable for displaying in the explorer/experimenter gui

---


### setSearch

```
public void setSearch(ASSearch search)
```

:   Sets the search method

    :   **Parameters:**: `search` - the search method with all options set.

---


### getSearch

```
public ASSearch getSearch()
```

:   Gets the search method used

    :   **Returns:**: the search method

---


### getCapabilities

```
public Capabilities getCapabilities()
```

:   Returns default capabilities of the classifier.

    :   **Specified by:**: `getCapabilities` in interface `CapabilitiesHandler` **Overrides:**: `getCapabilities` in class `SingleClassifierEnhancer`
    :   **Returns:**: the capabilities of this classifier **See Also:**: `Capabilities`

---


### buildClassifier

```
public void buildClassifier(Instances data)
                     throws java.lang.Exception
```

:   Build the classifier on the dimensionally reduced data.

    :   **Specified by:**: `buildClassifier` in class `Classifier`
    :   **Parameters:**: `data` - the training data **Throws:**: `java.lang.Exception` - if the classifier could not be built successfully

---


### distributionForInstance

```
public double[] distributionForInstance(Instance instance)
                                 throws java.lang.Exception
```

:   Classifies a given instance after attribute selection

    :   **Overrides:**: `distributionForInstance` in class `Classifier`
    :   **Parameters:**: `instance` - the instance to be classified **Returns:**: the class distribution **Throws:**: `java.lang.Exception` - if instance could not be classified successfully

---


### graphType

```
public int graphType()
```

:   Returns the type of graph this classifier
    represents.

    :   **Specified by:**: `graphType` in interface `Drawable`
    :   **Returns:**: the type of graph

---


### graph

```
public java.lang.String graph()
                       throws java.lang.Exception
```

:   Returns graph describing the classifier (if possible).

    :   **Specified by:**: `graph` in interface `Drawable`
    :   **Returns:**: the graph of the classifier in dotty format **Throws:**: `java.lang.Exception` - if the classifier cannot be graphed

---


### toString

```
public java.lang.String toString()
```

:   Output a representation of this classifier

    :   **Overrides:**: `toString` in class `java.lang.Object`
    :   **Returns:**: a representation of this classifier

---


### measureNumAttributesSelected

```
public double measureNumAttributesSelected()
```

:   Additional measure --- number of attributes selected

    :   **Returns:**: the number of attributes selected

---


### measureSelectionTime

```
public double measureSelectionTime()
```

:   Additional measure --- time taken (milliseconds) to select the attributes

    :   **Returns:**: the time taken to select attributes

---


### measureTime

```
public double measureTime()
```

:   Additional measure --- time taken (milliseconds) to select attributes
    and build the classifier

    :   **Returns:**: the total time (select attributes + build classifier)

---


### enumerateMeasures

```
public java.util.Enumeration enumerateMeasures()
```

:   Returns an enumeration of the additional measure names

    :   **Specified by:**: `enumerateMeasures` in interface `AdditionalMeasureProducer`
    :   **Returns:**: an enumeration of the measure names

---


### getMeasure

```
public double getMeasure(java.lang.String additionalMeasureName)
```

:   Returns the value of the named measure

    :   **Specified by:**: `getMeasure` in interface `AdditionalMeasureProducer`
    :   **Parameters:**: `additionalMeasureName` - the name of the measure to query for its value **Returns:**: the value of the named measure **Throws:**: `java.lang.IllegalArgumentException` - if the named measure is not supported

---


### getRevision

```
public java.lang.String getRevision()
```

:   Returns the revision string.

    :   **Specified by:**: `getRevision` in interface `RevisionHandler` **Overrides:**: `getRevision` in class `Classifier`
    :   **Returns:**: the revision

---


### main

```
public static void main(java.lang.String[] argv)
```

:   Main method for testing this class.

    :   **Parameters:**: `argv` - should contain the following arguments: -t training file [-T test file] [-c class index]


---


|  |  |  |
| --- | --- | --- |
| **Overview** \| **Package** \| **Class** \| **Tree** \| **Deprecated** \| **Index** \| **Help** \| **Weka's home** | |  |
| **PREV CLASS**   **NEXT CLASS** | **FRAMES**    **NO FRAMES**     **All Classes** |  |
| SUMMARY: NESTED \| FIELD \| CONSTR \| METHOD | DETAIL: FIELD \| CONSTR \| METHOD |  |


---
